# Mental health experiences in men’s elite-level cycling: A qualitative study of retired professional riders

**DOI:** 10.1192/j.eurpsy.2026.11514

**Published:** 2026-07-31

**Authors:** A. Smith, J. Grana, S. Hachen, E. Schollerer, A. Buadze, M. Liebrenz

**Affiliations:** 1 Department of Forensic Psychiatry, University of Bern, Bern; 2 Psychiatric Hospital, Zurich, University of Zurich, Zurich, Switzerland

## Abstract

**Introduction:**

Evidence has highlighted sizeable prevalence rates for mental illnesses in men’s elite-level cycling, especially for eating disorders, depression, and other conditions. In these contexts, distinctive sport-specific risk factors linked to performance pressures, sociocultural determinants, and weight concerns can increase athlete vulnerabilities. However, the complexity of individual riders’ lived experiences, particularly regarding career transitions and team support systems, remains largely underexplored.

**Objectives:**

This qualitative investigation aimed to address this gap by examining mental health experiences amongst former elite-level cyclists to identify potential stressors and the level of available support to inform evidence-based interventions.

**Methods:**

Semi-structured interviews were conducted with thirteen former male professional cyclists (Pro Continental-World Tour level; ages 29-44; minimum seven years professional experience). Thematic analysis following was employed to identify recurring patterns and core themes across the participants’ responses.

**Results:**

The analysis revealed five primary thematic domains: (1) persistent concerns associated with contract insecurity and short-term employment structures; (2) systematic pressures to maintain sub-optimal body weight; (3) inadequate mental health infrastructure within team environments compounded by confidentiality concerns that deterred help-seeking; (4) widespread use of sleep medications and benzodiazepines during competition; (5) potential psychological issues during career transition characterised by identity disruption, financial instability, and absence of structured support mechanisms.

**Conclusions:**

These findings identify substantial mental health challenges embedded within the culture and structures of elite men’s cycling, which potentially affect rider wellbeing. The difficulties cyclists face during retirement further expose the absence of coordinated transition pathways. Addressing these gaps may require wider reforms, including independent and confidential mental health services, evidence-based guidelines on medication use, and structured transition programmes that extend beyond performance-oriented support.

**Disclosure of Interest:**

None Declared

